# An integrated machine learning-based model for joint diagnosis of ovarian cancer with multiple test indicators

**DOI:** 10.1186/s13048-024-01365-9

**Published:** 2024-02-20

**Authors:** Yiwen Feng

**Affiliations:** 1 Departments of Obstetrics and Gynecology, Shanghai General Hospital, Shanghai Jiaotong University School of Medicine, Shanghai, 200080 P.R. China; 2 Jiuquan Hospital, Shanghai General Hospital, 200003 Shanghai, China

**Keywords:** Ovarian cancer, Machine learning, Test indicators, Diagnosis, Discriminative ability

## Abstract

**Objective:**

To construct a machine learning diagnostic model integrating feature dimensionality reduction techniques and artificial neural network classifiers to develop the value of clinical routine blood indexes for the auxiliary diagnosis of ovarian cancer.

**Methods:**

Patients with ovarian cancer clearly diagnosed in our hospital were collected as a case group (*n* = 185), and three groups of patients with other malignant otolaryngology tumors (*n* = 138), patients with benign otolaryngology diseases (*n* = 339) and those with normal physical examination (*n* = 92) were used as an overall control group. In this paper, a fully automated segmentation network for magnetic resonance images of ovarian cancer is proposed to improve the reproducibility of tumor segmentation results while effectively reducing the burden on radiologists. A pre-trained Res Net50 is used to the three edge output modules are fused to obtain the final segmentation results. The segmentation results of the proposed network architecture are compared with the segmentation results of the U-net based network architecture and the effect of different loss functions and region of interest sizes on the segmentation performance of the proposed network is analyzed.

**Results:**

The average Dice similarity coefficient, average sensitivity, average specificity (specificity) and average hausdorff distance of the proposed network segmentation results reached 83.62%, 89.11%, 96.37% and 8.50, respectively, which were better than the U-net based segmentation method. For ROIs containing tumor tissue, the smaller the size, the better the segmentation effect. Several loss functions do not differ much. The area under the ROC curve of the machine learning diagnostic model reached 0.948, with a sensitivity of 91.9% and a specificity of 86.9%, and its diagnostic efficacy was significantly better than that of the traditional way of detecting CA125 alone. The model was able to accurately diagnose ovarian cancer of different disease stages and showed certain discriminative ability for ovarian cancer in all three control subgroups.

**Conclusion:**

Using machine learning to integrate multiple conventional test indicators can effectively improve the diagnostic efficacy of ovarian cancer, which provides a new idea for the intelligent auxiliary diagnosis of ovarian cancer.

## Introduction

Magnetic resonance imaging (MRI) provides multiparameter multiplanar imaging with excellent soft-tissue resolution and has become the imaging method of choice in the preoperative evaluation of ovarian cancer. Accurate segmentation of ovarian cancer from MRI is important for subsequent diagnosis and treatment [1, 2]. Segmentation of ovarian cancer mainly relies on manual outlining by radiologists, which is not only subjective but also time-consuming and labor-intensive [3]. Therefore, a reliable method for automatic segmentation of ovarian cancer MRI images is urgently needed. In addition, different imaging equipment and imaging parameters have a great impact on the imaging quality, which may result in different density distributions of ovarian cancer in MRI of different patients, which further increases the difficulty of segmentation.

MRI ovarian cancer tumor auto-segmentation has been poorly studied and can be grouped into the following two broad categories.

1. Non-neural network approach [4] proposed a knot ovarian cancer tumor level set segmentation method [5] proposed a regional growth-based ovarian cancer tumor segmentation method, which is not able to achieve fully automatic segmentation [6] proposed a super-pixel-based tumor segmentation method for ovarian cancer. The method is not fully automatic, and some cases need to be manually excluded due to the presence of uterine fibroids, which leads to similar DCE-MRI performance.
2. Neural network-based method [7] proposed an ovarian cancer segmentation method, which uses convolutional neural networks (CNNs) to achieve the segmentation of the tumor region [8–10].

Res Net was proposed by [11] that The network architecture of this class adopts a deep residual connection framework to solve the training difficulties and accuracy degradation problems of deep networks, and generates network models with strong feature expression capabilities, which are widely used in tasks such as segmentation and detection, but so far Res Net has not been applied to segmentation of ovarian cancer tumor magnetic resonance images.

Image classification, object recognition, and semantic segmentation are just a few of the many uses for the ResNet-50 model in computer vision. Some examples of the numerous possible uses are listed above. Its wide acceptance can be attributed to the fact that it serves its intended purpose and is also reasonably accurate.

The excellent performance on many different computer vision benchmarks attests to ResNet-50's precision [12]. For example, using the massive ImageNet dataset, which contains photographs labeled in over a thousand different ways, it achieved an accuracy of 95.6%. The reliability of the system was evaluated using this data set.

In order to train a model for a new type of task, transfer learning can be used to leverage a previously-trained model. The ResNet-50 network is suitable for this technique. Ultimately, the goal of this training is to improve the model's performance on the novel task.

Aiming at the MRI ovarian cancer tumor segmentation challenges and the shortcomings of existing segmentation methods, FCN based automatic segmentation network for ovarian cancer MRI images is proposed [13]. In the proposed network, a ResNet50 with strong feature expression capability is used for feature extraction, and a pre-training strategy is used in the study in order to reduce the demand for data volume.

Dynamic contrast-enhanced magnetic resonance imaging (DCE-MRI) has advanced to the point where it is not only useful for detecting breast cancer but also for monitoring its development and understanding where tumors are located [14–17]. However, not only is this a laborious process, but the accuracy also relies on the radiologist's level of experience and training. However, radiomic data are used in medical imaging and have the potential to extract non-obvious features of sickness. Better patient diagnosis and care is possible with this data. Hard-coded features, known as 'radiomics,' reveal crucial data about the disease at the photographed site [18]. Deep learning methods, on the other hand, such convolutional neural networks (CNNs), can be trained to automatically learn features from a dataset. In the field of medical imaging, in particular, CNNs perform better than methods based on hard-coded characteristics. However, by combining the strengths of these two types of features, the accuracy is greatly improved, which is very crucial in the medical industry. Using DCE-MRI data, this work details the creation of a stacked ensemble of gradient boosting and deep learning models for breast cancer classification. Breast DCE-MRI image pixel data is used to calculate radiomics, which are then added into the model. Radiomics was applied to the factor analysis before the model was trained to enhance the quality of the feature set and eliminate any characteristics that were not helpful [19, 20].

Existing ovarian cancer markers have limited sensitivity and specificity, and it is difficult for a single indicator to accurately reflect the complexity of ovarian cancer pathogenesis [21]. The malignant risk transport algorithm adopts the strategy of multi-indicator joint diagnosis, i.e., multi-parameter model, and calculates the ROMA index by combining glycan antigen 125 (CA125) and human epididymis secretary protein 4 (HE4), whose sensitivity in ovarian cancer diagnosis is 89%, and its specificity is 79% [22]. With the development of artificial intelligence, machine learning is gradually applied to data analysis in the medical field [12]. Clinical laboratories can provide rich disease data resources for machine learning, and machine learning methods, by virtue of their powerful autonomous learning ability, extract the implied rules or models between test indicators and diseases from them, and construct a more complex and sophisticated multi-parameter combination approach [23]. Starting from the established test indicators of ovarian cancer patients, machine learning methods such as principal component analysis, genetic algorithms, neural networks, etc. are integrated to try to provide accurate and convenient decision support for ovarian cancer diagnosis.

The rest of the paper is organized as follows: Subject and methods section describes the subjects and methods; The research subjects come from the otolaryngology department, oncology department, and physical examination center of our medical center section details the proposed network model; Selection of indicators section presents the analysis of results; Demographic information section presents the discussion of results and Test items section concludes the paper.

## Subjects and methods

### The research subjects come from the otolaryngology department, oncology department, and physical examination center of our medical center

1. Case group: First diagnosed as primary ovarian cancer from January 2008 to December 2017, 185 female patients, aged 16–83 years.
2. Control group:569 cases of non-ovarian cancer, female patients or physical examiners in the same period. In order to enhance the ability of the model to identify related diseases, 3 control subgroups were set up. ①138 cases of other malignant otolaryngology tumor, aged 27–85 years.② 92 cases of physical examiners without otolaryngology benign or malignant diseases and apparently healthy, aged 20–84 years.

The above study subjects did not undergo surgery, radiotherapy or medication before enrolment, did not have cardiac, pulmonary, hepatic, renal and other organ insufficiency, did not have hematology diseases, were not combined with acute or chronic infectious diseases, and excluded pregnancy. Diagnosis was confirmed by clinical signs, imaging and pathology before the study subjects were discharged from the hospital; if there was no pathological examination, it must be confirmed by two or more imaging evidence (CT, MRI, B-mode ultrasound, etc.) in agreement.

### Selection of indicators

The collection was completed by the medical record information mining software independently developed by Shanghai Le jiu Medical Technology Co.

#### Demographic information

Epidemiological investigations have identified a variety of risk factors for ovarian cancer, of which three demographic characteristics that are well supported in the literature and well documented in the medical records were selected: age, whether or not menopausal, and number of pregnancies.

#### Test items

Twenty-eight hematology indexes with a clear relationship with ovarian cancer (including those related to the degree of progression or metastasis of ovarian cancer) were selected and classified into 6 categories: (1) 8 items of tumor markers, including carcinoembryonic antigen 125 (CA 125), squamous cell carcinoma-associated antigen (SSC); (2) 4 blood cell-related parameters, (3) 5 items of sex hormones, including β Chorionic Gonadotropin ( β- HCG), estradiol (E2), progesterone (P), luteinizing hormone (LH), follicle stimulating hormone (FSH); (5) 5 biochemical indicators, including albumin (Alb), globulin (Glo), prealbumin (PA), C-reactive protein (CRP), and fasting plasma glucose (FPG); (6) There are four indicators of lipid metabolism, including triacylglycerol (TG).

HE4 is an important ovarian cancer-related tumor marker that has been widely used in the clinic in recent years, and is superior to CA125 in some cases. The HE4 program was launched in our hospital in 2013.The patients who were admitted to the hospital at an earlier time in this study lacked the results of the HE4 test, and therefore HE4 was not included in the index collection.

### Statistical analyses

SPSS 25.0 was used to create a database and statistically describe the data. Programming with Matlab 2014a was used for z-score standardization, principal component analysis, BP neural network training and testing. ROC analysis was performed with Medcalc 15, $AU{C^{ROC}}$ and two-by-two pairwise comparisons were performed using the De Long test. Differences were considered statistically significant at *P* < 0.05.

## The designed network model

### Preprocessing

Although the images of all patients were acquired by the same device, the density distribution of T2W-MRI still varies from patient to patient. For this reason, these images are normalized as follows.

1 $${I}_{norm} = \frac{I - {I}_{min}}{{I}_{max} - {I}_{min}}$$

In order to investigate the effect of the degree of class imbalance on segmentation, the normalized images were cropped to obtain ROIs of different sizes covering the tumor region. To prevent network overfitting, the training dataset was augmented. The training images were rotated at an angle of 90° and 180°, respectively, and flipped up and down to make the training dataset five times the original size.

### Network architecture

**Fig. 1:**
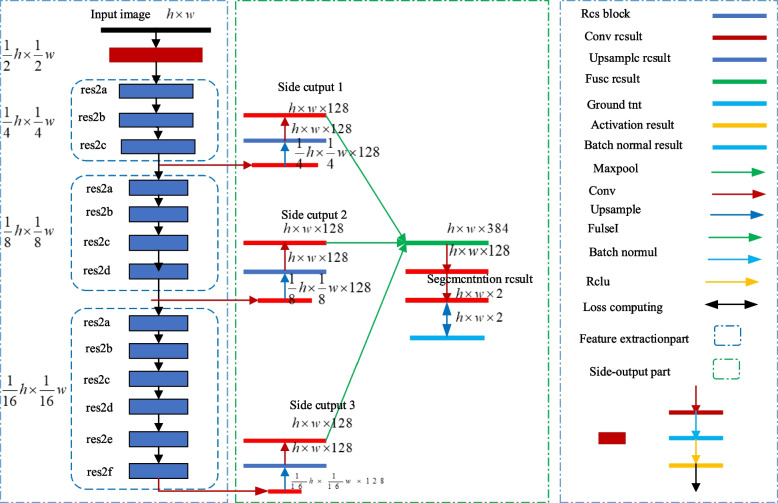
Architecture of the network

The network architecture consists of two parts: feature extraction and edge output, see Fig. 1. In the feature extraction part, Res Net50 is used as the basic network architecture, and the feature map containing multi-scale information is obtained through the middle layer of this network. Since the feature maps produced by layers with a step size of 32 or higher are too small in size, which can lead to too blurred results after interpolation, the last three residual modules of ResNet50, the pooling layer and the network fully connected layer are removed for this reason.

In order to guide multi-scale feature learning, the edge output module is added to the last layer of the 3rd, 7th and 13th residual modules of ResNet50, which enables multi-scale feature extraction from image data, taking into account both image details and overall information. However, since the sizes of these feature maps are not the same, it is not possible to utilize the multi-scale information by direct fusion. It is necessary to introduce a deconvolution layer in the edge output module to Upsampling the feature map according to the size of the input image, so that feature map fusion is possible. Therefore, before the inverse convolution, all the feature maps are subjected to a dimensionality reduction operation through a convolutional layer, so that the number of channels of the feature maps is reduced to 128, which reduces the computational cost while preserving the feature information. Ultimate the fused feature maps are dimensionality reduced by two convolutions to get the final segmentation result.

### Loss function

Denote the training dataset as $S = \left\{\left({X}_{i}, {Y}_{i}\right), i =\mathrm{1,2},\dots ,N\right\}$, where the gold standard corresponding to the input image ${X}_{i}$ and denote the network layer parameters as $W$.

In ovarian cancer tumor segmentation, there is a class imbalance problem, where the number of pixels in the non-tumor region of a T2W-MRI slice is much higher than the tumor region. For this reason a class-balanced cross-entropy loss function is used in an attempt to solve the problem. It balances positive and negative samples by introducing a class-equilibrium weight, defined as follows.

2 $$\begin{array}{c}\mathrm{CBCELoss }= -\beta \sum\limits_{{j\in Y}_{+}}{{\text{log}}}_{e} \mathrm{Pr }\left({y}_{j} =1\left|X;W\right.\right)\\ -\left(1 - \beta \right) \sum\limits_{{j\epsilon Y}_{-}}{{\text{log}}}_{e} {\text{Pr}} \left({y}_{j} =0\left|X;W\right.\right)\\ \beta = \left|{Y}_{-}\right|/\left|Y\right|, 1 - \beta = \left|{Y}_{+}\right|/\left|Y\right|\end{array}$$

The class probability $\mathrm{Pr }\left({y}_{j} =0\left|X;W\right.\right) \in \left[\mathrm{0,1}\right],n =\mathrm{0,1}$ is given by the final classifier, which evaluates the probability that the input pixel $j$ belongs to class $n$.

In order to investigate the ability of the class-balanced cross-entropy loss function to solve the class imbalance problem, the classical cross-entropy loss function and the Dice loss function are introduced for comparison.

### Training and testing of the model

The study population was divided into two parts by stratified random sampling, defining 2/3 of them as the training group and the other 1/3 as the testing group. The training process of BP neural network was optimized by embedding genetic algorithm, and parameters such as the number of neurons in the hidden layer, connection weights and thresholds of the BP neural network were adaptively determined based on its parallel stochastic search ability. Finally, the trained model was used to predict the diagnostic results of the test group (the output value was a continuous variable between 0 and 1), and the ROC curve of the test group was plotted, and the diagnostic efficacy of the model was evaluated by the three indexes, namely sensitivity, specificity and area under the ROC curve ${AUC}^{ROC}$, which corresponded to the highest point of the Youden index, with the value of 01 being the main The ${AUC}^{ROC}$ value was the main evaluation index.

## Experiments and analysis of results

### Realization

Implement the proposed network architecture using the publicly available Kera library. The device used is equipped with a workstation of NVIDIA GTX1080 Ti GPU, with a CUDA version of 8.0 [24].

### Assessment criteria

The Dice similarity coefficient is a spatial overlap metric that can be used to assess the similarity of overlap between segmentation results and the gold standard. Its value varies from 0 to 1, where 0 indicates that there is no overlap between the segmentation results and the gold standard, and 1 indicates that they are completely overlapped.

3 $$DSC = \frac{2TP}{2TP +FP +FN}$$

Sensitivity, which can also be referred to as the true positive rate, takes a value in the range of 0 ~ 1. The larger the value, the closer the segmentation result is to the gold standard.

4 $$Se = \frac{TP}{TP +FN}$$

The specificity, sometimes called the true negative rate, also ranges from 0 to 1. The larger the value, the more similar the segmentation results are to the gold standard, and conversely, the less similar they are.

5 $$Sp = \frac{FN}{TN +FP}$$

The hausdorff distance calculates the similarity between two sets of point sets and can be used to assess the difference between the segmentation results and the gold standard. The smaller the hausdorff distance, the closer the segmentation results are to the gold standard.

6 $$\text{HD}\left(P,G\right)=\text{max}\left(\underset{p\in P}{\text{max}}\underset{g\in G}{\text{min}}\Arrowvert p-g\Arrowvert,\underset{g\epsilon G}{\text{max}}\underset{p\epsilon P}{\text{min}}\Arrowvert g-p\Arrowvert\right)$$

Where: $\Vert \bullet \Vert$ is the Euclidean distance function; *P* is the segmentation result; *G* is the gold standard.

### Results and analyses

Segmentation performance of the U-net based network and the proposed network, where the optimal parameters of the U-net based network are set based on their paper. In the experiments, the network uses a class-balanced cross-entropy loss function, and the training and test images used are cropped from normalized images, both containing tumor tissue, and have a size of 96 × 96 pixels. A total of four different metrics were used to evaluate the segmentation results, namely DSC, sensitivity, specificity and HD. Table 1 shows the comparison of the segmentation results of the two networks.

**Table 1:** Segmentation results of the two networks

Evaluation indicators	U-net-based network	Proposed network
DSC/%	67.59 ± 7.70	83.62 ± 10.25
Se/%	83.52 ± 19.14	89.11 ± 10.21
Sp/%	91.20 ± 7.22	96.37 ± 3.60
HD	16.81 ± 7.35	8.50 ± 5.17

In Table 1, the average DSC, average sensitivity, average specificity, and average HD of the segmentation method proposed in this paper reached 83.62%, 89.11%, 96.37%, and 8.50, respectively, which improved the Dice similarity coefficient, sensitivity, and specificity by 16.03%, 5.59%, and 5.17%, respectively, and reduced the HD compared to the segmentation method based on the U-network by 8.31 [25].

**Fig. 2:**
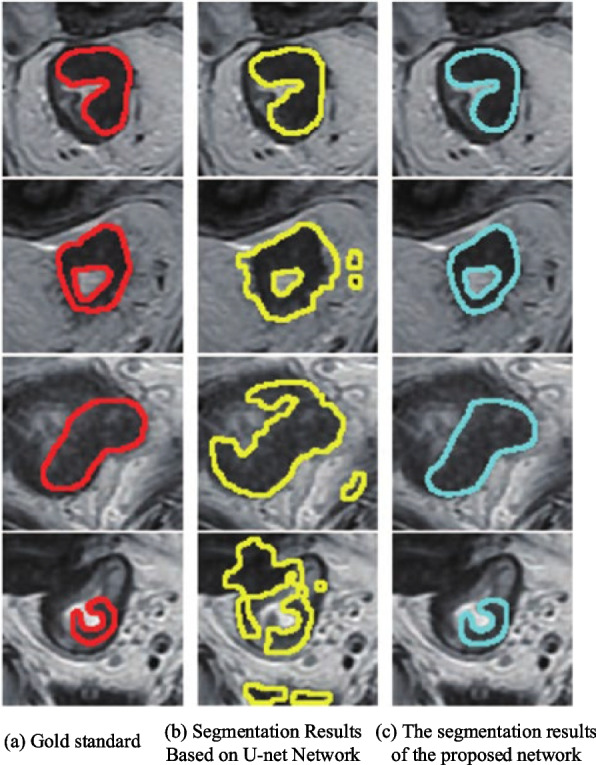
T2W-MRI segmentation results of ovarian cancer tumors

Figure 2 demonstrates the T2W-MRI segmentation results of four ovarian cancer tumors. Where (a) is the gold standard, (b) and (c) are the segmentation results of the U-net based network and the proposed network, respectively. Row 1 shows a simple example in which the tumor region is clearly different from normal tissue, and the segmentation results of both networks are similar to the gold standard. The tumor in row 2 is ring-shaped, and the proposed network is able to accurately segment this shape of ovarian cancer, while the U-net-based network shows over-segmentation. Rows 3 and 4 are cases where the demarcation line between the tumor region and the normal tissue is blurred, and the over-segmentation phenomenon of the U-net-based network is obvious, and some normal tissue regions are incorrectly segmented as tumor regions, while the proposed network does not show mis-segmentation. Compared with the U-net-based network, Fig. 2 visually shows the effectiveness of the proposed network for T2W-MRI segmentation of ovarian cancer tumors, especially for the complex cases with blurred boundaries between tumor regions and normal tissues, the proposed method is more effective.

**Fig. 3:**
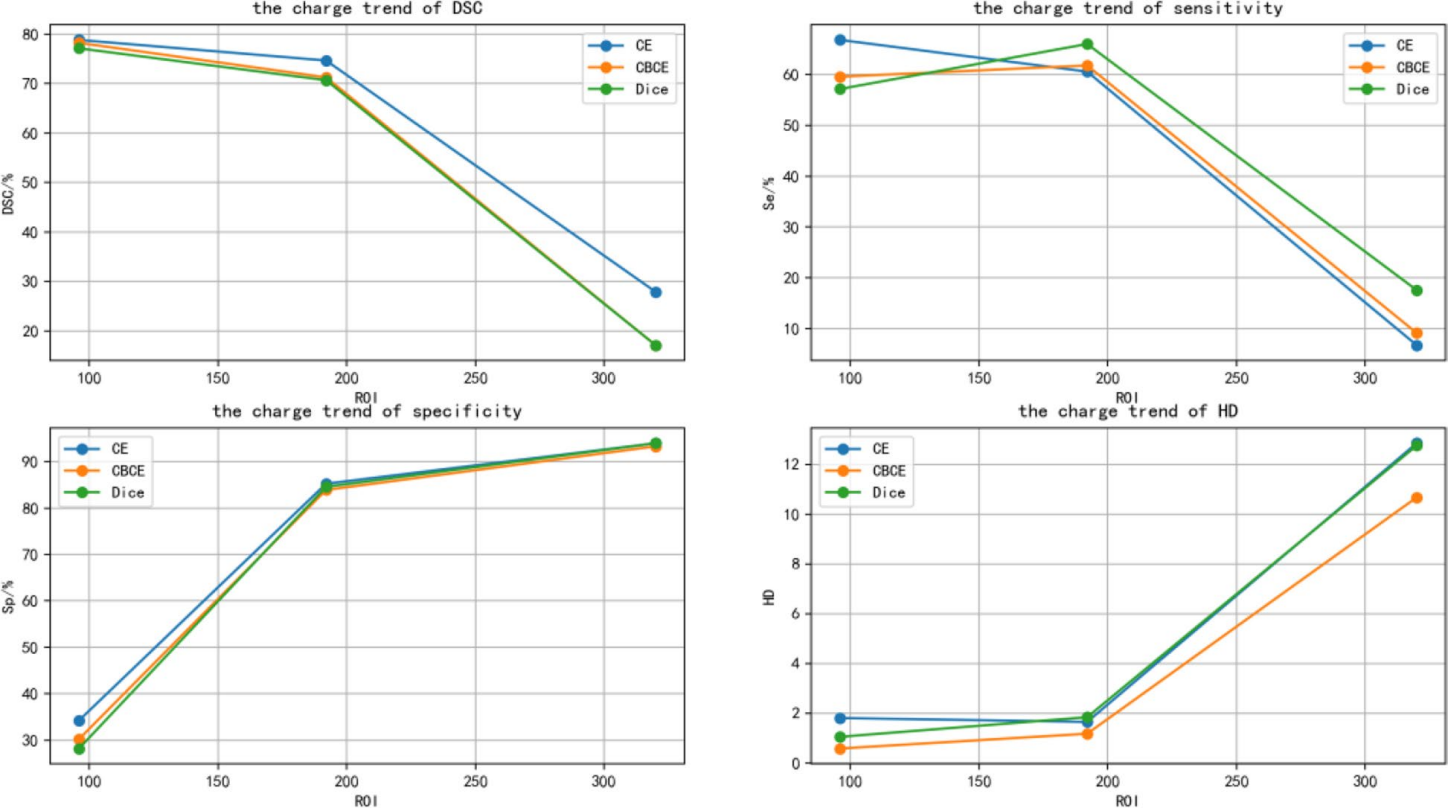
Trend of model segmentation results with image size based on different loss functions

The images were first cropped to 96 × 96, 192 × 192, and 320 × 320pixel sizes, respectively. Then 3 different types of networks are constructed based on the 3 different loss functions described earlier, and each type of network can be divided into 3 networks based on the size of their input images. In this way 9 different networks are designed, and then these networks are trained using cropped images of corresponding sizes. The results are shown in Fig. 3 that all sizes of input images, there is no significant difference between the 3 loss functions. No matter which loss function is based on, the network segmentation performance decreases as the input image size increases. The optimal segmentation results are obtained when the ROI region size is 96 × 96 pixels.

### Feature dimensionality reduction of test metrics

**Fig. 4:**
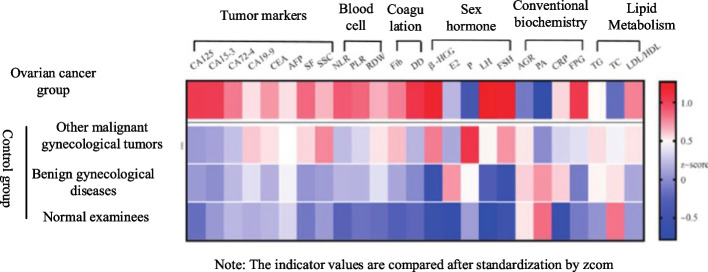
Heat map of test indicator levels in ovarian cancer group and control subgroups

The levels of the indicators in the ovarian cancer group were compared with those in the control group, and the results are shown in Fig. 4. The gradient change of the color blocks from blue to red in the heat map corresponds to the average level of the indicators from low to high. This shows that the average levels of E2, P, AGR, PA, and TC in the ovarian cancer group were lower than those in the control group, while other indicators were higher than those in the control group as a whole. Principal component analysis was used to filter the redundant features in the data and refine the core features to explain the differences between the samples. The three principal components with the largest eigenvalues were selected to represent the information of the original indicators, as shown in Table 2. The expressions of the first three principal components are.

**Table 2:** Eigenvectors corresponding to the first 3 principal components

Index	Number	Principal component 1	Principal component 2	Principal component 3
CA125	A1	0.616	0.237	0.043
CA15-3	A2	0.662	0.077	-0.047
CA72-4	A3	0.658	0.099	0.011
CA19-9	A4	-0.017	0.010	0.088
CEA	A5	0.191	0.005	-0.047
AFP	A6	-0.083	0.173	-0.023
SF	A7	0.263	0.260	0.215
SSC	A8	0.111	-0.015	-0.046
NLR	B1	0.037	-0.034	0.037
PLR	B2	0.173	0.646	-0.008
RDW	B3	0.687	0.082	0.094
Fib	C1	0.647	0.100	0.208
DD	C2	0.005	0.041	-0.076
$\beta$-HCG	D1	-0.225	-0.030	0.028
E2	D2	0.693	0.117	0.148
P	D3	0.648	0.125	0.220
LH	D4	-0.296	-0.267	-0.237
FSH	D5	0.080	0.704	0.030
AGR	E1	0.510	0.307	0.217
PA	E2	0.143	-0.059	0.014
CRP	E3	-0.162	-0.086	-0.849
FPG	E4	0.267	0.600	0.124
TG	F1	0.277	0.613	0.008
TC	F2	-0.124	-0.299	-0.079
LDL-C/HDL-C	F3	0.145	-0.004	0.854

7 $$\begin{array}{c}P_1=0.616A_1+0.662A_2+\cdots+0.145F_3\\P_2=0.237A_1+0.277A_2+\cdots-0.004F_3\\P_3=0.043A_1+0.047A_2+\cdots-0.854F_3\end{array}$$

**Fig. 5:**
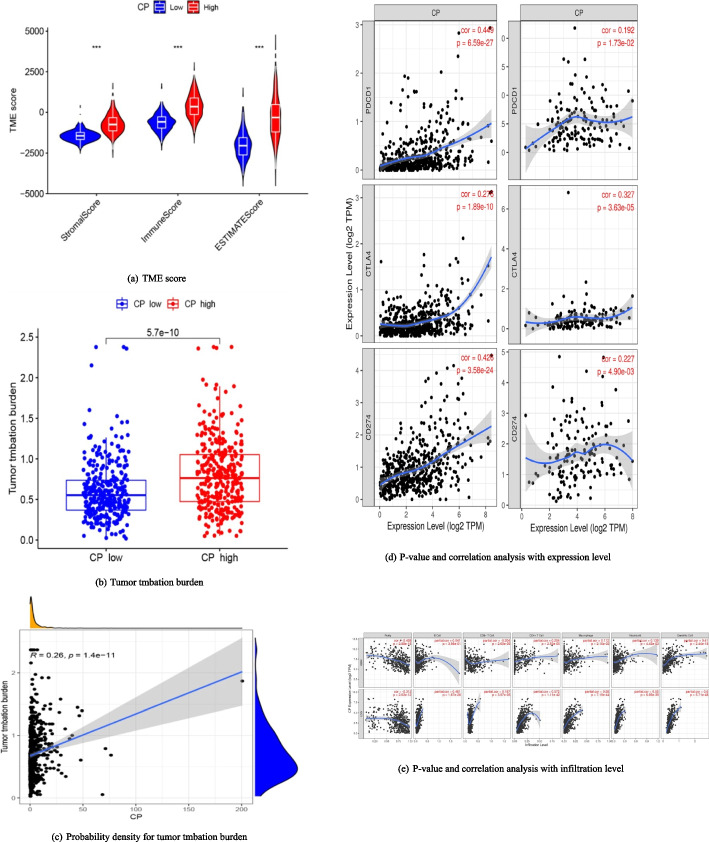
Distribution of the ovarian cancer group and the control group in the space of the first 3 principal component components

The larger the absolute value of the weight coefficient before the test indicator, the larger its contribution to the principal component. As can be seen from the expression of the principal components, principal component 1 mainly reflects the levels of CA125, CA15-3, CA72-4, and E2 and P, which can be categorized as gynecological tumors markers and hormone levels. By analogy, principal component 2 reflects glucose and lipid metabolism, and principal component 3 reflects inflammatory status. The principal component scores for each sample were calculated for multidimensional data visualization, see Fig. 5. Observe the subgroups of the three-dimensional scatters, it was found that these principal components demonstrated the differences between the ovarian cancer group and the control group more clearly.

### Evaluation of machine learning model training and diagnostic performance

The error curves converge quickly to a near-optimal solution, and have remained stable since then, indicating that the genetic algorithm optimizes the training parameters to achieve the desired results.

**Fig. 6:**
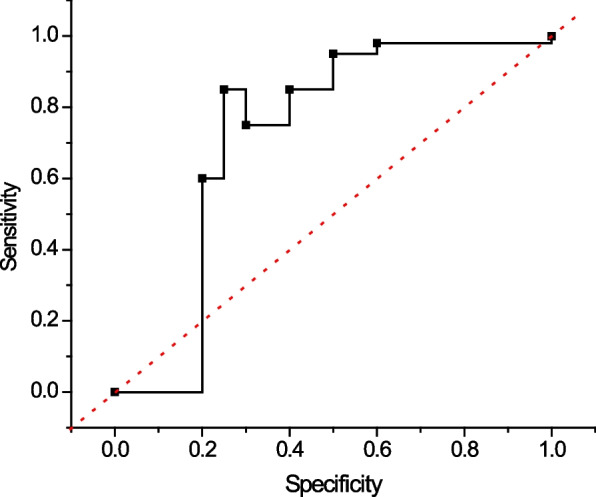
Diagnostic efficacy test for ovarian cancer

Blind prediction of the test group (62 ovarian cancer samples and 191 control samples) using the model constructed for the training group (123 ovarian cancer samples and 378 control samples) described above was compared with the diagnostic efficacy of CA125 in the test group, as shown in Fig. 6. The ${AUC}^{ROC}$ of the machine learning diagnostic model versus single CA125 was 0.948 and 0.746 (*P* < 0.01), the sensitivity was 91.9% and 74.2%, and the specificity was 86.9% and 73.3%, respectively, which significantly improved the diagnostic efficacy of ovarian cancer in combination with a multiple-indicator machine learning model compared with the traditional CA125.

### Diagnostic efficacy of models distinguishing between different stages of ovarian cancer and individual control subgroups

**Fig. 7:**
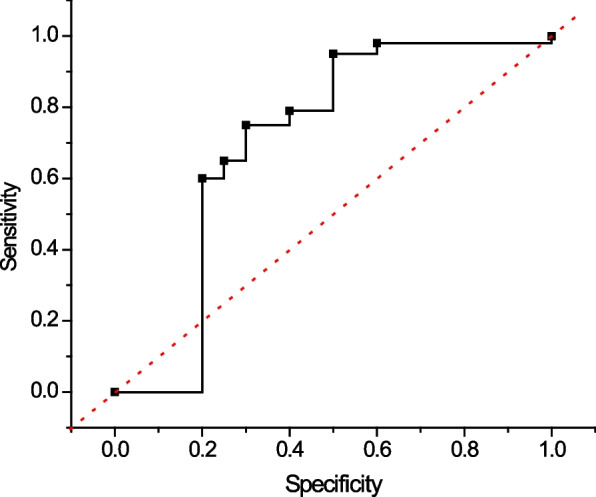
Diagnostic efficacy of ovarian cancer and various control subgroups

After verifying the total diagnostic efficacy of distinguishing ovarian cancer from the overall control group, to further investigate the generalization ability of the model in testing the change of the population type, the diagnostic efficacy of the model in distinguishing ovarian cancer in early stage (FIGO stage I-II) and advanced stage (FIGO stage III-IV) from the overall control group was compared, and as shown in Fig. 7. As shown in Fig. 7, the sensitivities and specificities of early and advanced stage ${AUC}^{ROC}$ were 0.944 and 0.955, respectively, and the sensitivities and specificities were very close to each other.

## Discussion

The secretive anatomical location of the ovary makes it difficult to implement intuitive examination methods such as tissue biopsy and luminal exploration, resulting in no improvement in the survival rate of ovarian cancer in China in the past 15 years [26]. Currently, there is no mature non-invasive diagnostic method for ovarian cancer, and vaginal ultrasound and serum CA125 are commonly used to screen for ovarian cancer in clinical practice, which are both plagued by the problems of low sensitivity and specificity. The search for a better multi-indicator combined diagnostic method has become the most promising breakthrough to improve the detection rate of ovarian cancer.

The booming development of artificial intelligence and machine learning technology provides a brand-new way for disease diagnosis [27]. As the most classical and active method in the field of machine learning, artificial neural network connects a number of neuron nodes with processing function according to a certain network structure by simulating the behavioral characteristics of the human brain, so that it can deal with fuzzy data or complex nonlinear mapping problems, and shows high inclusiveness to data noise caused by inconsistency of examination equipment and pathophysiological fluctuations. A number of studies on the use of machine learning methods such as neural networks for assisted diagnosis and grading of cancer have emerged in the medical community, and in general have demonstrated superior performance to traditional multivariate statistical classifications such as Logistic Regression Analysis and Fisher's Discriminant Analysis [28]. In this study, in order to simplify the input features of the neural network, the principal component analysis, which has a better effect of feature dimensionality reduction processing, is chosen as the predecessor system. At the same time, in order to solve the shortcomings of BP neural network, such as slow training speed and easy to fall into the local minimum, the step of genetic algorithm optimization is added. The above improvements make the total diagnostic performance of the integrated machine learning model reach a high standard with satisfactory results.

In selecting the indicators to be included in the model, this study followed the principles of economic practicality and localization. In recent years, some markers for novel ovarian cancer have been proposed at home and abroad, but most of them are histological in nature, with high testing costs and complex data analysis, and are far from forming a streamlined test like routine test items, thus lacking clinical practicality [29]. In contrast, the results of routine clinical test indicators are easy to obtain, facilitate inter-laboratory comparison, and meet the requirements of saving medical resources.

The demographic characteristics included in the model have all been documented to support their association with ovarian cancer. Although the exact cause of ovarian cancer is not fully understood, multiple risk factors have been identified through numerous epidemiological studies. The age distribution of ovarian cancer shows a certain pattern, with a rapid increase in ovarian epithelial cancer after the age of 40 years, with a peak age of 50–60 years and a gradual decline by the age of 70 years. Statistics show that the incidence of ovarian cancer in women of childbearing age gradually decreases as the number of pregnancies increases, while childless women are more likely to develop ovarian cancer. In addition, delayed menopause is also a risk factor for ovarian cancer [28]. The prevailing explanation is that the above demographic risk factors are often accompanied by persistent ovulation or increased ovulation, which leads to ovarian epithelial damage and prolonged mitogenic stimulation, which in turn induces malignant transformation of epithelial cells.

The test indicators included in the model were rich in information and covered a variety of physiological perspectives. 3 principal components distilled most of the characteristics of the 28 test indicators, which were basically in line with the medical community's knowledge of ovarian cancer. The relationship between gynecological tumors markers and sex hormones and ovarian cancer, as reflected in principal component 1, has been very clear and does not need to be repeated. Blood glucose and lipid metabolism reflected in principal component 2 are emerging hotspots in the field of ovarian cancer research. Poor blood sugar control increases the risk of cancer, and many studies have found that metformin, the first-line drug for diabetes, can have a therapeutic effect on ovarian cancer [29]. It is widely believed that diabetes induces ovarian cancer by releasing excessive insulin and disturbing the balance of sex hormones. In recent years, some cellular mechanisms and metabolic pathways of hyperglycemia carcinogenesis have been proposed one after another, opening up a new way of thinking for the prevention and treatment of ovarian cancer. More and more evidence demonstrate that obesity and lipid metabolism disorders are important risk factors for ovarian cancer and can promote ovarian cancer metastasis, and obesity is accompanied by increased TG and LDL-C/HDL-C. In order to meet the needs of rapid growth of cancer cells, a large amount of TC is consumed for the synthesis of new cell membranes, which may result in lower TC in ovarian cancer patients. Inflammation, which is mainly reflected by principal component 3, is ranked as the seventh most important biological feature of malignancy [30]. Inflammatory environment is inextricably linked with tumor development, providing advantageous conditions for tumor growth, invasion and metastasis with inflammatory environment. Therefore, in the clinical auxiliary diagnosis and differential diagnosis of ovarian cancer by means of tumor markers, it is recommended to refer to blood glucose, lipid metabolism, and CRP, NLR, RDW, and other indicators for evaluating inflammatory response.

The setting of the control group is a key factor in determining the performance of the diagnostic model. Considering that CA125, as the first marker of ovarian cancer, has increased in many gynecological benign diseases (Adenomyosis, endometriosis, gynecological inflammation, etc.) and some malignant tumor diseases (fallopian tube cancer, endometrial cancer, cervical adenocarcinoma, etc. Three control subgroups of patients with benign gynecological diseases and normal physical examination. Compared to similar studies that only use benign ovarian tumors or healthy women as controls, the control group in this study covers a wide range of diseases, undoubtedly improving the diagnostic difficulty of the model and being closer to clinical needs. It is worth noting that the model's ability to identify other malignant gynecological tumors is slightly weaker than the other two control subgroups. This model maintained a certain degree of differentiation ability in all three control subgroups, overcoming the shortcomings of CA125 in the differential diagnosis of related diseases. Similarly, considering that CA125 is not significantly expressed in early ovarian cancer patients, validation of the early and late stages populations has demonstrated that this model effectively compensates for the insufficient diagnostic ability of CA125 in early ovarian cancer.

## Conclusion

In this study, an ovarian cancer diagnostic model integrating multiple machine learning is established. The mainstream neural network diagnostic model is improved, and the principal component analysis and genetic algorithm are used to solve the two difficult problems of feature downgrading and training parameter optimization, respectively, to improve the learning ability and classification accuracy of the model. On the one hand, the secondary development of existing data resources is carried out to explore the diagnostic use of many routine test indicators to achieve a high degree of synergy and fusion; on the other hand, three control subgroups are set up to enhance the discriminative ability of the model for related diseases. The model has been tested and shown to significantly improve the diagnostic efficacy of ovarian cancer, with less interference from the stage of ovarian cancer and the type of control subgroups. There is still a long way to go for the clinical translation of the research results, which not only requires the medical units to dock the data mining system and cloud platform to provide the underlying technical support for the model, but also requires the expansion of the training sample size and the inclusion of more test indexes, such as HE4, and even the imaging data at a later stage in order to promote the iterative upgrading of the diagnostic performance of the model.

## Data Availability

The experimental data used to support the findings of this study are available from the Corresponding author upon request.
